# Editorial: Interdisciplinary approaches in veterinary sciences after COVID-19

**DOI:** 10.3389/fvets.2024.1361813

**Published:** 2024-01-16

**Authors:** Ariel L. Rivas, Stephen D. Smith

**Affiliations:** ^1^Center for Global Health, Internal Medicine, School of Medicine, University of New Mexico, Albuquerque, NM, United States; ^2^Geospatial Research Services, Ithaca, NY, United States

**Keywords:** infectious diseases, new therapies, zoonosis, medical geography, decision-making

At the end of December, 2023, the *Knowledge of Science*™ reported at least 657,000 publications that refer to COVID 19. It has been claimed that never in the history of science was so much published so fast on a single topic (1). Such an unprecedented development has generated numerous new challenges that can be synthesized with one question: how to summarize a vast and diverse body of published science into a publication that gives a synoptic view?

To answer that question, two different research styles should be reconciled, which involve specialized (uni-disciplinary) and inter-/transdisciplinary knowledge, respectively (2). Other problems affecting knowledge integration include: (a) reporting specialized research that is not necessarily accessible to readers unfamiliar with the specific field; (b) the fact that any selection of specialized studies is bound to be long and, therefore, difficult to be read; and (c) the local relevance of topics, which may apply to one place but not necessarily everywhere. While hundreds of systematic reviews on the COVID-19 related literature have been published (3) and many of them have been conducted within the context of veterinary medicine (4), the prompt applicability of such research, within Veterinary Medicine, remains unexplored. Given such challenges, this Research Topic Issue was designed to (i) generate a concise list of studies, (ii) follow a logic and structure that integrated two or more topics, disciplines, professions and/or technologies; and (iii) provide global perspectives.

Accordingly, ten studies are reported here, which involve authors and reviewers from more than twenty countries. These studies attempt to provide information potentially complementary and applicable. This Research Topic covers four focus areas, which refer to: zoonoses, new tests and therapies, geography and epidemiology, and social-biological sciences.

## Zoonoses

Focusing on potential reservoirs of infectious (zoonotic or epizootic) diseases, three studies conducted in the United States, the Netherlands and Georgia investigate domestic carnivores (Hecht et al.; Fischer et al.) and ruminants (Rivas et al.) with or without human data. The overall purpose of these studies is to expand the target of veterinary practices to address not only diseased individuals and populations but interactions that may include other species.

## New tests and therapies

To better detect and/or treat infectious diseases, new molecular tests or therapies (including acoustic pulse technology) are described by Indian and Israeli researchers (Das et al.; Blum et al.). The focus of these studies is to illustrate expanded or alternative approaches that may detect infectious diseases earlier or treat such diseases more effectively.

## Medical geography

Geography and epidemiology are integrated in two studies that include authors from Mexico, Chile, Spain, United States and Uruguay (Hoogesteyn et al.; Picasso-Risso et al.). These studies shed light on how the properties of geographical data may influence disease dissemination.

## The educational/policy-making continuum

The abundance and complexity of novel research may require new educational programs that explicitly include economic (cost/benefit-oriented) decision-making. That is conveyed from the perspective of German, Chinese, and Kenyan viewpoints (Becker et al.), economics (Yu et al.), and policy-making (Sitawa et al.).

The previous description of these studies is only partial because these works refer to many fields, theories or applications. For example, Picasso-Risso et al. use geographical data within the context of Network Theory. Addressing a topic of global relevance in policy-making (antimicrobial resistance), the possible use of therapies that do not utilize antibiotics is explored by Blum et al.. An example of an anticipatory study of wildlife species that may host potential vectors of emergent diseases (such as malaria induced by parasites previously found in non-human species) is offered by Das et al.. Integrating economics with control of zoonoses, Rivas et al. compare the cost-effectiveness of two approaches that utilize geo-referenced data. The fact that the prompt dissemination of these topics and technologies may require new educational configurations–including non-presential formats–is explored and evaluated by Becker et al..

Together, it is expected that this material may provide a guide to consider when developing new educational and research programs. The editors thank the participation of all authors and reviewers.

## Author contributions

AR: Conceptualization, Writing – original draft, Writing – review & editing. SS: Conceptualization, Writing – original draft, Writing – review & editing.
